# Comparison of different images in gross target volume delineating on VX2 nasopharyngeal transplantation tumor models

**DOI:** 10.7150/jca.36076

**Published:** 2020-01-01

**Authors:** Zhang-Qiang Xiang, Saber Imani, Yue Hu, Rui-Lin Ding, Hao-Wen Pang, Yue Chen, Shao-Zhi Fu, Fang Xie, Wen-Feng He, Qing-Lian Wen

**Affiliations:** 1 Department of Oncology, The Affiliated Hospital of Southwest Medical University, Luzhou, Sichuan, China (Z-QX, SI, YH, R-LD, H-WP, S-ZF, FX, W-FH, Q-LW).; 2 Department of Nuclear Medicine, The Affiliated Hospital of Southwest Medical University, Luzhou, Sichuan, China (YC).

**Keywords:** Nasopharyngeal carcinoma, Gross tumor volume, Magnetic resonance imaging, Computed tomography, ^18^F-FLT PET/CT, ^18^F-FDG PET/CT.

## Abstract

**Background:** To determine the optimum conditions for diagnosis of nasopharyngeal carcinoma, we established VX2 rabbit model to delineate gross target volume (GTV) in different imaging methods.

**Methods:** The orthotopic nasopharyngeal carcinoma (NPC) was established in sixteen New Zealand rabbits. After 7-days inoculation, the rabbits were examined by CT scanning and then sacrificed for pathological examination. To achieve the best delineation, different GTVs of CT, MRI, ^18^F-FDG PET/CT, and ^18^F-FLT PET/CT images were correlated with pathological GTV (GTVp).

**Results:** We found 45% and 60% of the maximum standardized uptake value (SUV_max_) as the optimal SUV threshold for the target volume of NPC in^ 18^F-FDG PET/CT and ^18^F-FLT PET/CT images, respectively (GTV_FDG45%_ and GTV_FLT60%_). Moreover, the GTV_MRI_ and GTV_CT_ were significantly higher than the GTVp (*P ≤* 0.05), while the GTV_FDG45%_ and especially GTV_FLT60%_ were similar to the GTVp (*R =* 0.892 and *R =* 0.902, respectively; *P* ≤ 0.001).

**Conclusions:** Notably, the results suggested that ^18^F-FLT PET/CT could reflect the tumor boundaries more accurately than ^18^F-FDG PET/CT, MRI and CT, which makes ^18^F-FLT PET-CT more advantageous for the clinical delineation of the target volume in NPC.

## Introduction

Nasopharyngeal carcinoma (NPC) is the most frequently diagnosed cancer in the Southeast Asia, Middle East, and North Africa, with estimated as 20-50 per 100,000 person-years in southern China 1, 2. According to Surveillance, Epidemiology, and End Results (SEER) program, the overall 5-year survival of NPC was > 80% after the radiotherapy, when diagnosed at the localized stage 2. Today, the modern radiographic modalities imaging and irradiation techniques, including magnetic resonance imaging (MRI) and computed tomography (CT) are among the more accurate distinction methods of retropharyngeal nodes and adjacent primary tumor of NPC 3-6. Since the resolution of MRI is higher than CT on soft tissue, the MRI imaging can be first choice for adequate targeting of NPC 7.

The high tumor proliferative rate and aggressive biological behavior of regional lymph nodes of NPC lead to a decrease in sensitivity of early diagnosis 8. Still, excellent metastases diagnosis of retropharyngeal lymph nodes metastases, cervical lymph nodes metastases, and distant metastases in patients with locoregionally advanced NPC is still poor 6, 9. Target delineation of the advanced-stage of NPC was routinely performed by the combination of CT and MRI to define the extension of the tumor involvement. It is becoming evident that positron emission tomography (PET) has recently emerged as a sensitive, specific and accurate s morphological imaging which can detect early malignant changes at the molecular level. More recently, functional imaging of PET/CT is widely used for the diagnosis and staging of many types of cancer, including breast cancer, lung cancer, head and neck cancer, and etc. 10-14. PET and CT combined imaging have reduced the inter- and intra-observer variation in contouring the gross tumor volume (GTV) compared to anatomic imaging techniques. Gradient-based segmentation method is one of the well-evaluated detection methods that have been developed to identify the NPC tumor based on the changes in the levels at the tumor border. The low spatial resolution, poor target delineation of distant metastases, and recurrence disease are among the most limitation in the PET images when enhanced with CT or MRI images 14. In fact, different imaging agents can produce accurate lesion data from different stages of the tumorigenesis, such as metabolism and proliferation. In this regards, ^18^F-Fluorothymidine (^18^F-FLT) and ^18^F-Fluorodeoxyglucose (^18^F-FDG) positron are well-adjusted PET/CT imaging techniques that were recombined by the National Comprehensive Cancer Network guideline 2012, for designing lung cancer target volumes 15. However, by the advent of FDG-PET, the therapeutic effectiveness of radiotherapy and earlier diagnosis of NPC has been improved, enthusiastically 8. PET-guided GTV delineation methods, ^18^F-FDG and ^18^F-FLT PET/CT are refelacted glucose metabolism. This could limit the distinguishing of the proliferation of tumor cells from inflammatory tissue. Also, the accuracy of the delineation using FLT PET/CT is not sufficiently validated according to the pathologic data and tumour type 16, 17.

Due to the notoriously narrow therapeutic margin, the target delineation in NPC is often challengeable. In microscopic imaging, the recognition of highly infiltrative is the first step of accurate delineation of the GTVs in high-risk site 18, 19. Recent articles indicate that high doses modalities imaging and irradiation techniques are required to achieve optimal levels of tumor control in individual patients 20. Among many clinical diagnosis and assisting tumor staging methods, ^18^F-FDG PET/CT and ^18^F-FLT PET/CT are the most acceptable and non-invasive procedures for the NPC staging and diagnosis 21. Despite many technological advances, the target GTVs delineation of ^18^F-FDG PET/CT and ^18^F-FLT PET/CT in NPC are still controversial.

With these assumptions and foreground, this study was aimed to compare the application value of ^18^F-FDG PET/CT and ^18^F- FLT PET/CT in the rabbit VX2 NPC model. Then, we tried to determine the best imaging method to delineate the GTVs of ^18^F-FDG PET/CT and ^18^F- FLT PET/CT image in NPC carcinoma.

## Materials and methods

### Animals and tumor model

Sixteen healthy New Zealand white rabbits, which are 8 females and 8 males, and their body's weight are between 2.8~4.0 kg (age 3-4 months), were obtained by the experimental animal center of Southwest Medical University (license number SCXK (Shanghai) 2013-2017 Shanghai Slack Experimental Animal Co., Ltd; SCXK2013-17). They were housed a standard laboratory cages under 20-22 °C, 50-60% relative humidity and 12 h light/ 12 h dark cycle (starting at 07.00 h and 19.00 h, respectively), with free access to food and water. The VX2 liver tumor-bearing rabbit model was obtained by he Ultrasonic Experimental Center of Chongqing Medical University (Chongqing, China).

### Establishment of the VX2 nasopharyngeal transplantation tumor model

Establishment of the rabbit VX2 transplantation model was performed according to the standard method 22, 23. The VX2 liver tumor-bearing rabbit was anaesthetized with 3% pentobarbital sodium (Solarbio science, Beijing, China) at a dose of 1 ml/kg body weight. The tumor-bearing rabbit was narcotized and fixed in the supine position, and was covered with a sterile drape exposing only the abdominal skin. After one week, the skin was incised and the tumor tissue stripped off into approximately 1.0×1.0 cm in a clean bench by using a sterile scalpel. The samples were placed in Hank's solution and with normal saline and then the tumor suspension were harvested after grinding using a 200-300 mesh sieve homogenizer homogenized with appropriate amount of 1% normal saline. The cell suspension (1-2×10^7^ cells/ml) was collected after centrifugal scrubber at 1500 g, for 5 min, by adding the proper amount of normal saline in the bottom of the cell suspension. Thereafter, suspended cells were cultured in FBS-free RPMI-1640 medium (RPMI, Hyclone, USA) supplemented with 1% penicillin-streptomycin (Sigma-Aldrich, St Louis, MO, USA). Cell cultures were incubated at 37 °C with 5% CO_2_ in a humidified incubator. The number of viable cells was counted by using the trypan blue exclusion test; the percentage of viable cells was > 93%.

For nasopharyngeal tumor cell implantation, rabbits were narcotized and fixed in the supine position. We selected the midpoint of the hyoid bone as the puncture point. By the guidance of CT, the needle immediately was inserted to reach the nasopharyngeal posterior wall adjacent to clivus. The rabbits received 0.5 ml VX2 cell suspension (1×10^7^ cells/ml) by slow submucosal injection of posterior pharyngeal wall so that the puncture depth was 3-5 mm below the surface of the skull plate. The injection was performed by using 1.0-ml syringe puncture needle (1.2 mm diameter) in duration of 3 minutes. After surgery, 40-60 million units of penicillin were given intramuscularly for 3 days.

After 7 days from implantation, a CT scan was performed to determine the tumor growth. When the mass in the nasopharynx found, the enhanced CT, MRI, ^18^F-FDG PET/CT, and ^18^F-FLT PET/CT examination of the rabbits were performed within 96 h. After the scanning, rabbits were immediately sacrificed and the tumor's tissue was fixed for further pathological assays.

### Contrast-enhanced CT and MRI imaging protocol

**Figure 1:**
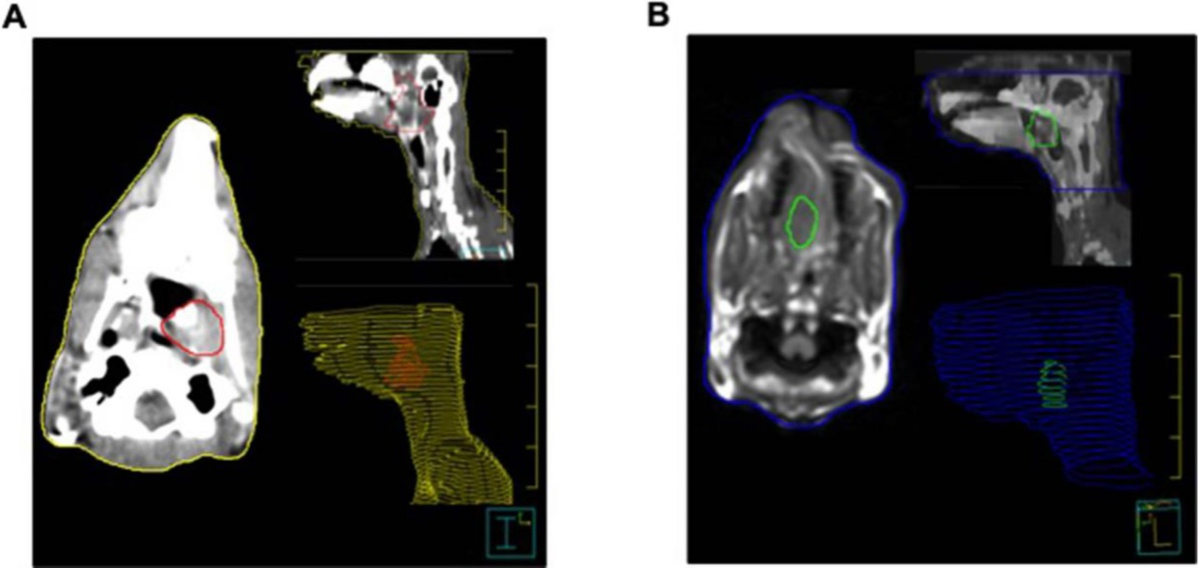
Representative images of GTV segmentation on contrast-enhanced CT images (**A**) and MRI (**B**) in a VX2 rabbit model with a nasopharyngeal carcinoma. The coronal images of the CT and MRI show GTV segmentation on the cavernous sinus and left orbit. The red zone on the panel A and green zone on panel B indicates the regions of NPC with less than 95% isodose level. Note that all outlines are manually drawn.

The anesthetized rabbits (n = 10) were fixed in the supine position on the home-made plastic fixing plate, and the head was fixed on a home-made foam holder. The rabbits underwent conventional cross-sectional scan with spiral CT (Light Speed ultra; GE Medical Systems, USA). The CT parameters included 2.5 mm slice thickness and 0.75 pitches were were checked routinely at 5 mm reconstructing interval. For contrast-enhanced CT imaging, 15 ml iohexol (300 mg I/ml, 1.5 ml/kg) was pumped into the rabbit through an ear marginal vein at a rate of 0.5 ml/s. Also, the MRI (Achieva 3.0T, Philips, Netherlands) studies were conducted by a head and neck coil, T1-weighted axial, sagittal scanning (TR2000 ms/TE20 ms with 3 mm slice thickness, 0.3 mm spacing), T2-weighted axial, sagittal scanning (TR3000 ms/TE80 ms with 3 mm slice thickness, 0.3 mm spacing). Post-contrast T1-weighted images were acquired 5 min after magnevist solution injection (Gd1DTPA, 0.1 mmol/kg). All images were transferred to a Pinnacle 8.0 Philips workstation. The GTVs in axial contrast-enhanced CT images (GTV_CT_, Fig. 1A) and MRI image (GTV_MRI_, Fig. 1B) were determined by two radiation oncologists (HW.P. and QL.W.). In details, T1-weighted contrast-enhanced MRI images in axial view and sagittal view of the tumor were delineated for the GTV_MRI_.

### PET/CT imaging protocol

The integrated PET and CT images were acquired on a hybrid PET/CT scanner (Gemini TF/16, Philips, Netherlands) at the Department of Nuclear Medicine, the Affiliated Hospital of Southwest Medical University. Before scanning, the rabbits were not given food and water for more than 12 h. Then, they were fixed in the same supine position as mentioned above. Next, 45.4-64.4 Mbq (the radiochemical purity > 95%) of ^18^F-FDG or 45.4-64.4 Mbq of ^18^F-FLT was injected intravenously through the ear marginal vein. Sixty min after the injection, the images were acquired. The PET/CT parameters included 120 KV, 80 mAS, 3 min/bed, 0.813 pitches, and a 3.8 mm reconstruction layer thickness. Data were reconstructed using an iterative reconstruction technique and attenuation correction derived from the CT data. The CT, PET, and fused PET/CT images were transmitted to an Extended Brilliance Philips workstation. The PET images were evaluated qualitatively for regions of focally increased metabolism. Increased uptake in the surrounding of the tissue was considered to characterize malignancy. For tumors visualized by PET, the region of interest was placed over the entire FDG- or FLT-avid lesion on all transverse planes in which the tumor appeared. The standardized uptake value (SUV) was calculated using the standard formula: SUV=A/(ID/BW), where, A is the decay-corrected tracer tissue concentration (Bq/g); ID, the injected dose (Bq) and BW, the body weight (g) of the rabbit 24.

**Figure 2:**
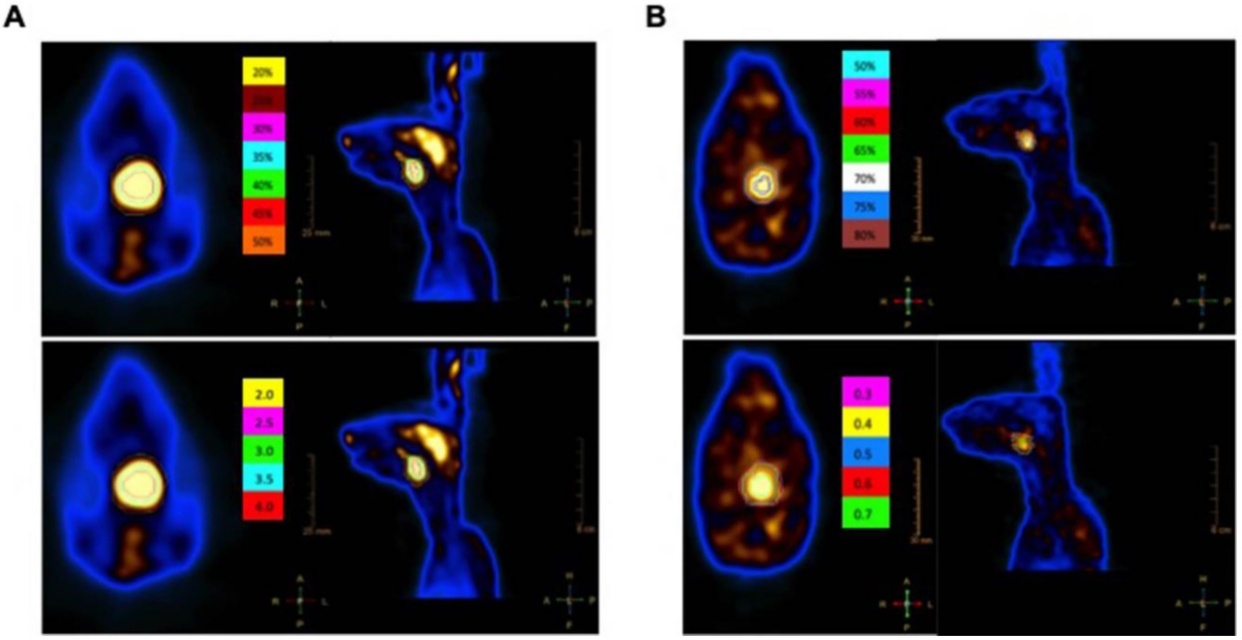
The GTV automatically segmented based on different SUV threshold values on ^18^F-FDG PET/CT **(A)** and ^18^F-FLT PET/CT **(B)**. The ^18^F-FDG PET/CT images (Panel A) GTV_FDG2.0_, GTV_FDG2.5_, GTV_FDG3.0_, GTV_FDG3.5_, and GTV_FDG4.0_ were automatically segmented on the PET images using gradient-based methods, fixed threshold values at 2.0, 2.5, 3.0, 3.5, and 4.0 SUV, respectively. The axial and coronal images of GTVs segmented based on different SUV threshold values at 50%, 55%, 60%, 65%, 70%, 75%, and 80% of SUVmax. Also, the axial and coronal MRI images of GTVs segmented based on different SUV threshold values at 0.3, 0.4, 0.5, 0.6 and 0.7 SUV, receptively (panel B).

In this study, the different kinds of GTVs were generated for each animal. For the ^18^F-FDG PET/CT images, GTV_FDG2.0_, GTV_FDG2.5_, GTV_FDG3.0_, GTV_FDG3.5_, and GTV_FDG4.0_ were automatically segmented on the PET images using gradient-based methods, fixed threshold values at 2.0, 2.5, 3.0, 3.5, and 4.0 SUV, respectively. GTV_FDG20%_, GTV_FDG25%_, GTV_FDG30%_, GTV_FDG35%_, GTV_FDG40%_, GTV_FDG45%_, and GTV_FDG50%_ were automatically segmented using the same method as above at fixed threshold values: 20, 25, 30, 35, 40, 45, and 50% of SUVmax (Fig. 2A). For the ^18^F-FLT PET/CT images, the GTVs were delineated in the same way as we described above in different fixed threshold values at 0.3, 0.4, 0.5, 0.6, and 0.7 SUV, respectively. Then, GTV_FLT50%_, GTV_FLT55%_, GTV_FLT60%_, GTV_FLT65%_, GTV_FLT70%_, GTV_FLT75%_, and GTV_FLT80%_ were automatically segmented using the same method as above at fixed threshold values: 50, 55, 60, 65, 70, 75, and 80% of SUVmax (Fig. 2B). The PET/CT images were reviewed by one experienced nuclear medicine physician (Y.C.) and two radiation oncologists (HW.P. and QL.W.).

### Pathological examination

For the histopathological staining, the rabbits were sacrificed immediately after completing all of the imaging examination. After division the bilateral cervical lymph node, the whole tumor and clivus of the rabbit was dissected. The pathology gross tumor volume (GTVp) was measured by the water immersion method. In this study, the GTVp was defined as the maximum major axis length and minimum minor axis length of the tumor in the target slice visible to the naked eye 22, 23. VX2 tumors were excised, and then fixed in a neutral formalin solution for 24 h. The specimens were stained by a standard hematoxylin and eosin (H&E) technique and examined by light microscopy. The pathological specimens were fixed with 10% formaldehyde solution, conventional dehydration, followed by paraffin imbedding. The best embedded-samples were cut into 4-µm-thick sections, and then stained the hematoxylin and eosin (H&E) for later light microscopy analysis (Nikon, Tokyo, Japan), linked to computerized image system (Image-Pro Plus V6.0, Silver Spring, MD) at 200x magnification. All procedures of tumours removeing from animals were performed by the same experienced surgical teams. All pathological examinations were assessed by two expert pathologists, independently (RL.D. and Y.H.) and any disagreements were resolved through a discussion.

### Statistical analysis

All quantitative data were transferred to Excel and the statistical analyses were computed with SPSS software for Windows (Version 21, SPSS Inc., Chicago, Illinois, USA). Data were presented as means ± Std. Deviation (SD) or median (range). The Wilcoxon signed rank test was used to compare the GTVp with GTV_CT_, GTV_MRI_, GTV_FDG_, and GTV_FLT_. The Spearman's rank correlation coefficient test was employed to determine the association between two variables. For all tests, *P <* 0.05 considered statistically significant. All charts were designed by Prism 5.0 (GraphPad, La Jolla, CA, USA).

## Results

### The establishment of animal model

Sixteen New Zealand white rabbits were used to determine VX2 nasopharyngeal transplantation tumor models. However, one rabbit died of massive hemorrhage due to the piercing of a large blood vessel during the establishment of the tumor model. After 7-day inoculation, the CT images were acquired on the remaining rabbits (n = 15) and nasopharyngeal masses were found in 13 of 15 rabbits (86.67%), which were later confirmed by pathological examinations. In this successful tumor model, 3 rabbits (20%) died from suffocation due to the oversize tumors, airway obstruction, and/or anesthesia-induced respiratory depression. Thus, the remaining 10 animals (76.92%) were eligible for the study to delineate the GTVs. This presentence of mortality during establishment of the VX2 transplantation model is in line with the results of previous studies 22.

### VX2 transplantation tumor growth and pathological finding

**Figure 3:**
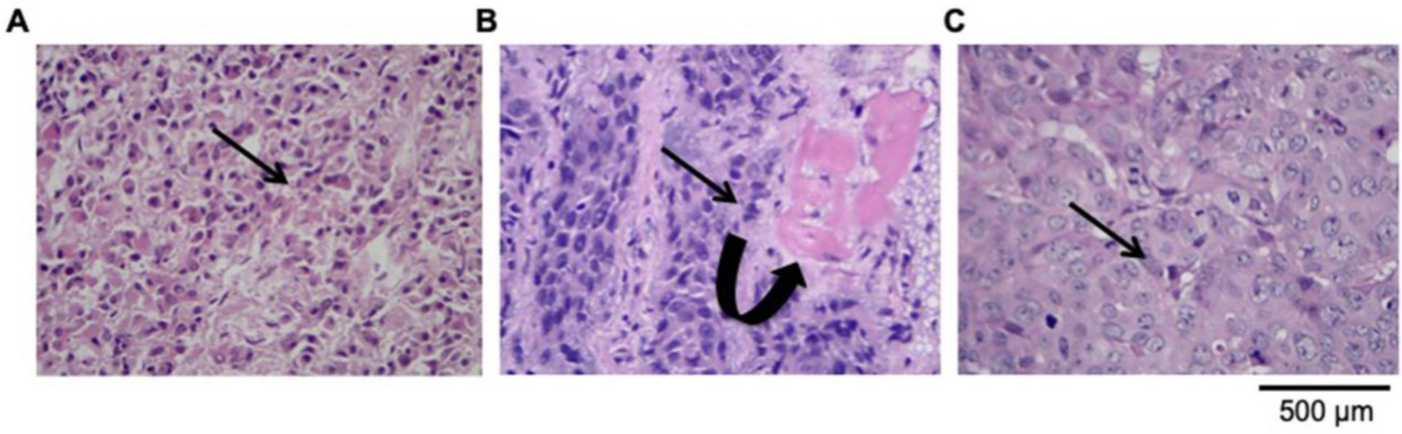
Microscopic pathological image of VX2 nasopharyngeal transplantation tumor metastasis (original magnification: ×400). **(A).** H&E-stained of poorly differentiated squamous cell carcinoma. **(B)**. Representative image of lymph node metastasis, **(C).** Representative image of pulmonary metastasis in VX2 nasopharyngeal transplantation tumor model. Microscopic pathology showed that tumor invasion exceeded the GTV visible to the naked eye and the tumor extent shown on the map (The curved arrow indicates the area of CTV_P_). The infiltrations of leukocytes and destroyed clivus cell were observed around the vessels (arrow pointed) as well.

The VX2 tumor is a transplantable rabbit squamous cell carcinoma, characterized by rapid tumor growth and early metastasis. To measure the growth and metastatic pattern, we acquired the CT images during the planning phase: 7, 14, 21, and 28 days after transplantation. On day 7 after inoculation, there was no difference in the tumor volume. However, the tumor's volume increased after 7 days (over time). The distinct morphology and growth pattern of VX2 transplantation cells suggested some differences in their malignant characteristics of the carcinoma cells. At 14 days from transplantation, the mean nasopharyngeal tumor volume reached 5.86 cm^3^ and the average diameter was 2.83 ± 0.56 cm in all rabbits. Subsequently, imaging examination and autopsy confirmed that the tumor exhibited a local aggressive behavior. To evaluate VX2 nasopharyngeal transplantation tumor metastasis, squamous cell carcinoma sections were stained with H&E and analyzed by using light microscopy. Observation of H&E-staining under a 100× microscope clearly demonstrated the tumor invasion in posterior pharyngeal wall and inflammatory cell infiltration in the necrotic zone. Poorly differentiated squamous cell carcinoma was found in orthotopic NPC, its cervical lymph nodes, metastatic lesions of brain and lung by H&E staining. Deformation of the cell structure and liquefaction necrosis in the GTV_p_ area is the remarkable histological finding (Fig. 3). As the Fig. 3A has shown, VX2 nasopharyngeal tumor poorly differentiated in squamous cell carcinoma structure with intensely stained nuclei, increased cell density, and some cells revealed pathological mitosis. By considering both viables and necrotic tumor tissues, the parapharyngeal space (10/10), clivus (6/10), and oropharynx (1/10) are among the most disoriented and depolarized structural. We found that the cervical lymph node metastasis is the most common metastasis pattern, with more than 60% of the rabbits (6/10; Fig. 3B). Moreover, 2 rabbits were confirmed with pulmonary metastasis (20%, Fig. 3C).

### Relationship between pathological GTV and imaging techniques GTV

**Figure 4:**
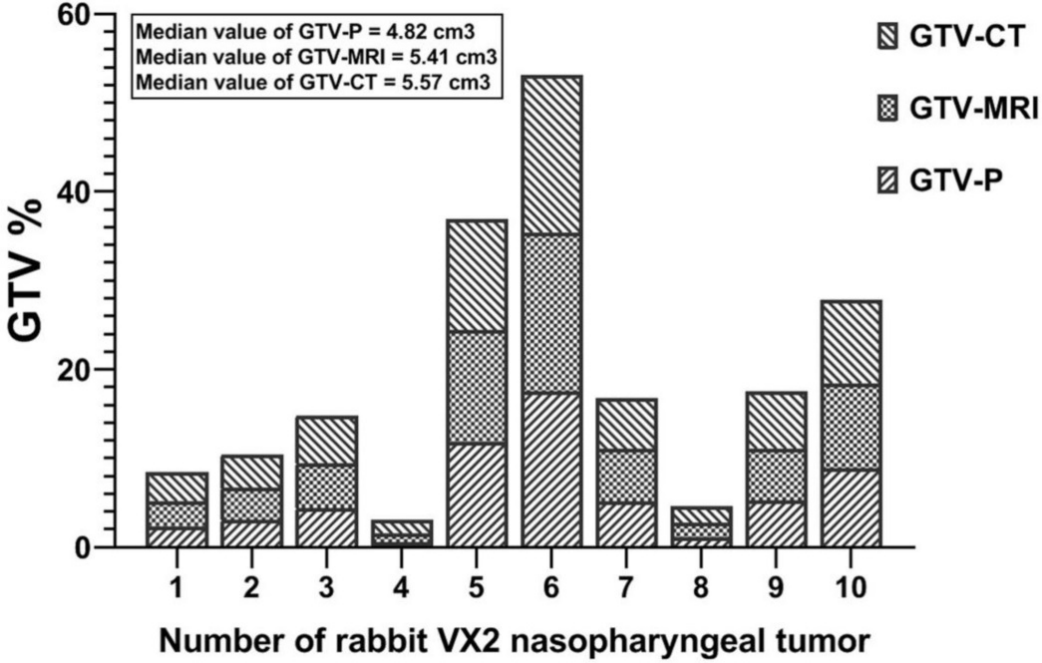
Comparison of pathological GTV** (**GTV_p_) and GTV of MRI (GTV_MRI_) and CT (GTV_CT_) in 10- VX2 nasopharyngeal transplantation rabbit.

The mean value of GTVp with GTV of MRI (GTVMRI) and CT (GTVCT) imaging are compared in Fig. 4. In details, three rabbit had upper GTV NPC (Case No: 5, 6, and 10), three had middle GTV NPC (Case No: 3, 7, and 9), and four had lower GTV disease (Case No: 1, 2, 4, and 8). The GTVP major ranged from 0.54 to 17.49 cm3 in the 10 rabbit NPC with VX2 tumor and the mean was 6.04 ± 5.04 cm3. Importantly, the GTV_MRI_ and GTV_CT_ showed an increasee in the GTV area; 1.12-fold increasing for GTV_MRI_ and 1.15-fold increasing for GTV_CT_. The mean value of the GTV**_CT_** was 6.77 ± 4.86 cm^3^; ranged 1.49-17.74 cm^3^. In GTV_MRI_ images the major value ranged from 1.02-17.91 cm^3^ and the mean was 6.56 ± 5.04 cm^3^ (Fig. 4).

### Comparison of the ^18^F-FLT and ^18^F-FDG uptakes in tumor-bearing rabbits

Table 1 compares the maximal uptake volumes of the ^18^F-FLT and ^18^F-FDG created as well as the results of the statistical comparison in the VX2 NPC rabbit. All rabbits with nasopharyngeal mass (n = 10) significantly increased the ^18^F-FLT and ^18^F-FDG uptake (*P <* 0.05). The SUV_max_ of the FDG was significantly higher than that of FLT (*P* = 0.004). According to Table 1, SUV_max_ of the ^18^F-FDG PET/CT and ^18^F-FLT PET/CT were 8.22 ± 4.95 (3.27-11.06) and 0.66 ± 0.57 (0.27-1.23), respectively. Furthermore, SUV_min_ of the FDG was also significantly higher than FLT (3.43 ± 1.70 vs. 0.20 ± 0.16; *P =* 0.011). Consequently, FLT uptake in regional lymph nodes of NPC is significantly lower than FDG uptake. As shown in Table 1, the pooled specificity was higher in the ^18^F-FDG PET/CT compared with the ^18^F-FLT PET/CT (0.769 versus 0.847). The diagnostic sensitivity of both ^18^F-FLT PET/CT and ^18^F-FDG PET/CT were 100% (10/10). Meanwhile, the highest sensitivity and specificity of the ^18^F-FLT PET/CT suggested that the FLT based PET/CT is more accurate and visualized in NPC diagnosis.

**Figure 5:**
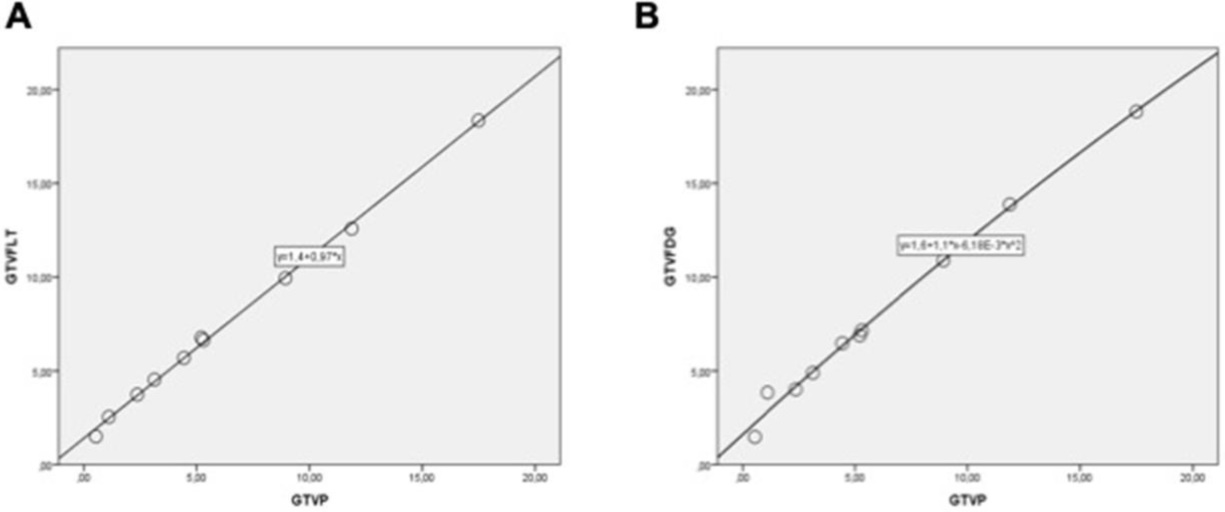
Pooled correlation between GTVp & GTV_FLT_ **(A)** and GTVp & GTV_FDG_ **(B).** Data indicate positive associations in phantom models with the Spearman's rank correlation coefficients. The mean of GTV is reported in the cm^3^. Each circles represents one VX2 nasopharyngeal transplantation rabbit (n = 10).

Pooled correlation coefficients between GTV_P_ and imaging techniques (GTV_FDG_ and GTV_FLT_) are shown in the Fig. 5. Despite of the small size of each sample, we found that the GTV_P_ ratios were strikingly correlated with the GTV_FLT_ (*R =* 0.902, *P =* 0.007; Fig. 5A) and GTV_FDG_ (*R =* 0.892, *P =* 0.005; Fig. 5B) in 10-VX2 nasopharyngeal transplantation rabbit, regardless of the different acquisition times. Totally, the significant correlation between the GTV_p_ with PET/CT imaging suggested the effective validity of PET/CT techniques for variety of different type of tumors. Conversely, due to the small size of cases, this result must be considered with restraint.

### The optimal SUV threshold of ^18^F-FDG PET/CT and ^18^F-FLT PET/CT images for target volume delineation

In order to identify the optimal SUV threshold of the ^18^F-FDG PET/CT and ^18^F-FLT PET/CT, different threshold values of GTV_FLT_ and GTV_FDG_ at different SUV were compared with the GTVp, as a standard GTV (Table 2).

According to the Table 2, there are differences between the created GTVs. While the differences between GTV_FLT_ vs. GTV_P_ and GTV_FDG_ vs. GTV_P_ appear to be larger, all the other differences were equal to or smaller than the spatial resolution of the GTV_P_ system (*P* < 0.05). The GTV_FLT0.6_, GTV_FLT55%_, and GTV_FLT60%_ were chosen to be compared with the GTVp, due to having more similarity to the GTVp than the other GTVs (*P* > 0.05). In other words, the difference between GTV_FLT0.6_, GTV_FLT55%_, and GTV_FLT60%_ with the GTVp was not statistically significant either (Figure S1). However, the GTV_FLT60%_ was closer to GTVp, accordingly the 60% of the SUV_max_ was assumed as the optimal SUV threshold for the^ 18^F-FLT PET/CT images to delineate the target volume of NPC (*P =* 0.36). In addition, the GTVs delineated on the ^18^F-FDG PET/CT in different threshold values are compared with GTVp in Figure S2. According the ^18^F-FDG PET/CT results; we found just three GTV_FDG_ are not significantly different from the GTVp; GTV_FDG4.0_, GTV_FDG45%_, and GTV_FDG50%_ (*P >* 0.05). Nevertheless, the GTV_FDG45%_ was much closer to GTVp, suggesting that 45% of the SUV_max_ could be used as the optimal SUV threshold for ^18^F-FDG PET/CT images in the delineation of the NPC target volume (*P =* 0.074).

### Comparison of MRI, CT, ^18^F-FDG PET/CT and ^18^F-FLT PET/CT in target volume delineation

**Figure 6:**
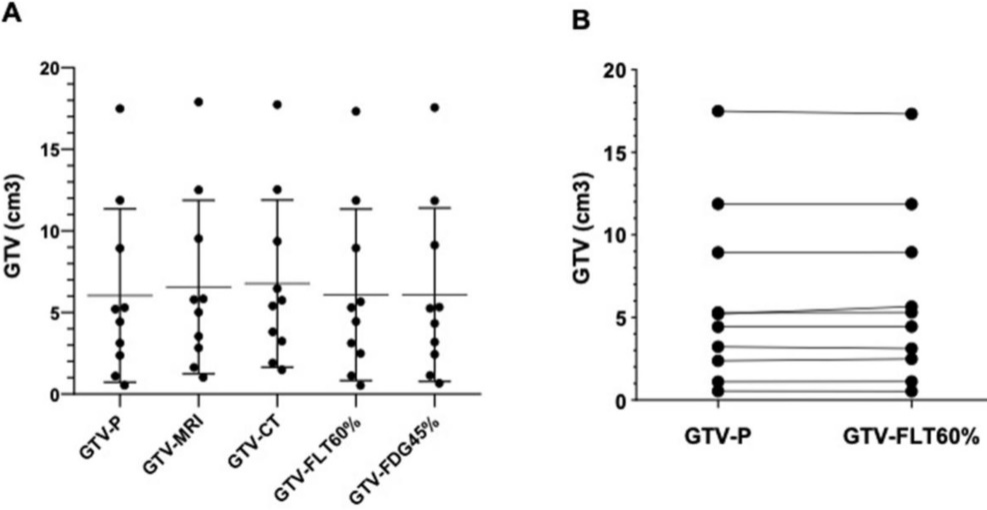
(**A**). Frequencies of the pathological GTV in comparison with different imaging methods: GTVCT, GTVMRI,GTVFDG, and GTVFLT. (**B**). Case-by-case comparison between 18F-FLT PET/CT images and pathology methods. The GTVFDG45% and GTVFLT60% segments are selected based on optimal SUV threshold values on 18F-FLT PET/CT and 18F-FDG PET/CT images. Data are representative charts or the percentages of an individual animal. The lines indicate median values for each group.

Here, we compared the GTV_FDG45%_, GTV_FLT60%_, GTV_MRI_, and GTV_CT_ with GTVp as a standard GTV (Fig. 6A). These comparisons showed no difference in the GTV_FDG45%_ (6.09 ± 5.04 cm^3^) and GTV_FLT60%_ (6.07 ± 4.99 cm^3^) in comparison with GTVp (6.04 ± 5.04 cm^3^; *P =* 0.103). These findinges are in line with the relationship between GTV_P_ and other imaging, GTV_CT_ and GTV_MRI_ (see the Fig. 4). In total, these results clearly showed that the PET/CT images were more suitable to delineate the target volume of NPC than the CT and/or MRI images. Furthermore, the difference between GTV_FDG45%_ and GTV_FLT60%_ was not statistically significant (*P >* 0.05). There was a cloth similarity between GTV numbers, measured in the ^18^F-FLT PET/CT images, and the pathology samples (mean number of GTV_FLT60%_ 6.078 cm^3^ vs 6.047 cm^3^ GTVp, *P =* 0.989, Fig. 6B). However, this similarity was predominately attributed in samples with very high GTV (> 7 cm^3^ GTV in both specimen of pathology and ^18^F-FLT PET/CT). In total, the GTV_FLT60%_ was the closest to the GTVp, suggesting that the ^18^F-FLT PET/CT images were more useful in target volume delineation of the NPC imaging.

## Discussion

To the best of our knowledge, this is the first report that evaluated accurate tumor volume delineation for the intrinsic character of PET/CT images in the NPC. In this investigation, we tried to determine the best imaging method for delineating the GTV of NPC. These findings indicate that a combination imaging of the PET and CT approach could potentially monitor metastasis in advanced NPC. Notably, our finding shows that GTV_FDG45%_ and GTV_FLT60%_ were a more effective and reasonable threshold value of ^18^F-FDG PET/CT and ^18^F-FLT PET/CT for target volume delineation in the VX2 nasopharyngeal transplantation tumor models. Furthermore, distribution of the GTV_FLT60%_ in GTV_P_ suggested that the ^18^F-FLT PET/CT images were more useful in target volume delineation of the NPC imaging. We established a rabbit VX2 nasopharyngeal transplantation model to replicate human NPC. New Zealand rabbits were relatively large in volume, easy to inoculate by puncturing and to perform experimental observations. We found that the tumors grew rapidly in the rabbit model, and gradually invaded the surrounding tissue; including parapharyngeal space, oropharynx, nasal cavity, and etc. Accordingly, the rabbit VX2 nasopharyngeal tumor model can simulate the whole process of the development of human NPC and provides a reasonable animal model for the follow-up experiments in target volume delineation.

Currently, radiotherapy has been widely accepted as a first-line and standard treatment for advanced NPC, by providing the superior dose of distributions and the better tolerated on NPC patients 25-27. The more precise the radiation technique becomes the more rigid the requirements are to precisely delineate the tumor. However, the target volume of NPC is so irregular, and the ccurate delineation of target areas is a prerequisite for precise radiotherapy plan. Thus, the delineation of the target volume is the key factor to determine the therapeutic effect as well as the reduction of the side effects of radiotherapy 28-30. The ^18^F-FDG, which reflects the of malignancy degree of the cells by glucose metabolism, is an acceptable imaging agent in clinical applications. However, the results are not accurate in FDG imaging, duo to the susceptible physiological activities of the body and also the activity of the lesion.

The ^18^F-FLT is a kind of thymine isomers, which is transported into cells by Na^+^ dependent, facilitates the diffusion and passive diffusion. The ^18^F-FLT PET/CT can show the proliferative activity of malignant tumors; so ^18^F-FLT imaging is more specifically than the ^18^F-FDG 29, 30.

Recently findings emphasize that the accuracy of the ^18^F-FLT PET/CT in distinguishing the tumor lesions and inflammatory is better than ^18^F-FDG PET/CT. In radiotherapy, the uptake of FLT is related to the proliferating cell nuclear antigen; thus the FLT can evaluate the efficacy of radiotherapy; so, this has more sensitive than FDG 31, 32.

Many semi-quantitative methods are widely used to define the threshold range of the SUV in the PET/CT image 14, 33, 34. Nevertheless, whether it is ^18^F-FDG or ^18^F-FLT PET-CT, the suitable SUV range to delineate the target volume has not yet been clearly defined 35. Enhanced CT, MRI, ^18^F-FDG PET/CT, and ^18^F-FLT PET/CT were separately used to scan the rabbit VX2 nasopharyngeal transplantation tumor model. GTV_FDG_ and GTV_FLT_ under the different setting conditions were delineated in the area of interest on the PET/CT images to compare them with GTVp, GTV_MRI_, and GTV_CT_ 35. We found that the optimal SUV thresholds of the ^18^F-FDG PET/CT and ^18^F-FLT PET/CT were 45% SUV_max_ and 60% SUV_max,_ respectively. Both of them are closer to the GTVp than the GTV_MRI_ and GTV_CT_. Both the CT and MRI methods are the first imaging choice for the staging of nasopharyngeal carcinoma 34, 36, 37. The results of many studies show that diagenetic accuracy of CT and MRI are equal in the detection of abdominal lymph node metastasis, intraluminal mass and pericardial effusion 38, 39. Our finsdings clearly show that both the CT and MRI were large to estimate the size of the lesion, which was consistent with previous findings by Chen *et al.*, (2013) 40. The present investigation results indicated that there was no significant difference between the GTV_FDG45%_ and GTV_FLT60%_ compared with the GTVp; thus ^18^F-FDG PET/CT and ^18^F-FLT PET/CT could both be useful tools for nasopharyngeal tumor target volume delineation under the optimal threshold setting. However, compared with the two methods, ^18^F-FLT PET/CT is more suitable to reflect the real tumor boundaries, avoiding the delineation of the target area involved in normal tissue, which could help in developing more accurate radiotherapy plans 41, 42. In this study, we found that the SUV_max_ and SUV_min_ of ^18^F-FLT PET/CT are significantly lower than the SUV_max_ and SUV_min_ of ^18^F-FDG PET/CT, which is consistent with the results of previous studies (Table 1) 43. These finding are in line with the results by study conducted by Shi *et al.* (2017) who used the 78% approach for GTV delineation potential to predict tumor regression in NPC patients 21. The preliminary results of that group reported that parameters of both FDG and FLT PET had medium to strong correlation with NPC tumor response after chemo-radiotherapy; thus this finding avoids the problem of incongruent imaging of tumor motion 21 .

It is accepted that VX2 transplantation rabbits with large and inhomogeneous tumors might be helpful in studies of dose escalation 44. A possible reason for such results is that the demand of tumor cell in the process of proliferation for thymidine is far lower than the glucose. Moreover, the level of thymine in animal serum is higher than that of human, and endogenous thymine can compete with FLT, as the FLT uptake in animals is lower than that of human beings.

Also, the results of our study suggested a better daignotsis and prognostic value of ^18^F-FLT than that in ^18^F-FDG, with a sensitivity of 100% and specificity of 84.7%. Highest sensitivity and specificity validated FLT as a promising PET radiopharmaceutical for early diagnosis, imaging proliferation, and monitoring tumor response of NPC 45. In this line, Hoshikawa *et al.,* (2011) and Chen *et al.,* (2005) confirmed that the uptake of ^18^F-FLT in tumor tissue is lower than ^18^F-FDG, its sensitivity and specificity is much higher than that of ^18^F-FDG 46, 47.

Although the SUV uptake of ^18^F-FLT PET/CT is low in our study, the image contrast is high enough to provide a "clean" display of tumor. It has been widely accepted that the brain uptake of ^18^F-FLT is low. Consequently, high T/N is beneficial for brain tumor imaging 32, 48. Although the focus of our study is on nasopharyngeal carcinoma, the low ^18^F-FLT uptake of brain tissue can provide a "clean" display of the background. Tumor tissue and normal brain tissue both have higher rates of glucose metabolism, which can result in unclear demarcation and difficult recognition in the two tissues in FDG PET. In general, FLT imaging can non-invasively reflect tumor proliferation at the molecular level. Additionally, it can distinguish inflammation from cancer, has higher tumor specificity than FDG, and can help in the differential diagnosis of tumor and anti-proliferative treatment efficacy evaluation and prognosis.

We should point out that there are some limitations in this investigation, to reflect whether the integration of FLT PET/CT into radiopharmaceutical of NPC can improve the treatment outcomes or not. Our study limitation includes the small sample size, different grading, and staging of the animal model. It is clear that tracers administered, time, and reconstruction algorithm needs to evaluate for standardization of a cutoff SUV on FLT PET/CT radiotherapy. As another limitation of the present study, the future learning needs to optimize the best SUV threshold of PET/CT with other pathology characterization of human NPC tumors, such as biological characteristic, tumor size, and motions of the lower esophagus. Thus, further researches with more human samples are needed to clarify the validity of our findings in the comprehensive clinical study.

## Conclusions

In conclusion, our study founds SUVmax cutoff of 45% on 18F-FDG PET/CT and SUVmax cutoff of 60% on 18F-FLT PET/CT as the closest estimation of GTV length for the target volume delineation in the NPC. Furthermore, ^18^F-FLT PET/CT by virtue of its ability to reflect the activity of cell proliferation, can use for the accrue delineation of the target area in the NPC. Our studies indicate that ^18^F-FLT PET/CT may be a promising imaging modality in the diagnosis and treatment of NPC.

## Supplementary Material

Supplementary figures.

## Figures and Tables

**Table 1:** The comparison of uptake value between ^18^F-FLT and ^18^F-FDG PET/CT in rabbit VX2 NPC model.

P-value	^18^F-FLT	^18^F-FDG	Parameters
0.004	0.66 ± 0.57 (0.27-1.23)	8.22 ± 4.95 (3.27-11.06)	ΣSUVmax
0.011	0.20 ± 0.16 (0.04-0.35)	3.43 ± 1.70 (2.13-5.13)	ΣSUVmin
0.007	0.61 ± 0.15 (0.25-0.76)	6.18 ± 1.25 (3.14-7.38)	ΣSUVmean
-	100	100	Sensitivity (%)
0.062	84.7	76.9	Specificity (%)

Abbreviations: ^18^F-FLT, ^18^F-Fluorothymidine; ^18^F-FDG, ^18^F-Fluorodeoxyglucose; SUV, standard uptake values; -, not available. 10 VX2 nasopharyngeal transplantation rabbits in each group. All data expressed as mean ± SD.

**Table 2:** Different SUV threshold of ^18^F-FDG PET/CT and ^18^F-FLT PET/CT images for target volume delineation.

		Mean ± SD	Median	Min	Max	P-value
GTV_p_	_-_	6.04±5.04	4.82	0.54	17.49	-
GTV_FDG_	FDG 2.0	7.83±4.99	6.68	1.47	18.82	0.005
	FDG 2.5	7.56±5.06	6.43	1.24	18.77	0.005
	FDG 3.0	7.01±5.09	5.75	1.19	18.53	0.005
	FDG 3.5	6.50±5.04	5.29	0.87	17.86	0.005
	FDG 4.0	6.10±5.05	4.81	0.66	17.59	0.059
	FDG 20%	7.92±4.93	6.61	1.87	18.72	0.005
	FDG 25%	7.46±5.03	6.29	1.53	18.58	0.005
	FDG 30%	7.08±5.07	5.84	1.14	18.35	0.005
	FDG 35%	6.55±5.10	5.27	0.87	17.72	0.005
	FDG 40%	6.11±5.05	4.78	0.67	17.59	0.047
	FDG 45%	6.09±5.04	4.79	0.66	17.56	0.074
	FDG 50%	5.92±5.02	4.44	0.61	17.34	0.059
GTV_FLT_	FLT 0.3	7.23±4.87	6.16	1.50	18.35	0.005
	FLT 0.4	6.87±4.95	5.73	1.25	18.22	0.005
	FLT 0.5	6.46±4.96	5.36	0.88	17.84	0.005
	FLT 0.6	6.05±5.04	4.85	0.52	17.52	0.169
	FLT 0.7	5.59±5.06	4.50	0.48	17.14	0.005
	FLT 50%	6.58±5.08	5.27	1.00	17.90	0.005
	FLT 55%	6.10±5.00	4.91	0.52	17.33	0.241
	FLT 60%	6.07±4.99	4.88	0.52	17.32	0.359
	FLT 65%	5.66±4.98	4.62	0.50	16.97	0.005
	FLT 70%	5.38±4.97	4.19	0.44	16.76	0.005
	FLT 75%	5.12±4.90	3.93	0.44	16.32	0.005
	FLT 80%	4.92±4.92	3.70	0.33	16.31	0.005

Abbreviations: ^18^F-FLT, ^18^F-Fluorothymidine; ^18^F-FDG, ^18^F-Fluorodeoxyglucose; SUV, standard uptake values. All data expressed as mean ± SD (range). The underlined threshold was selected to the future assay, as a best-matched- delineation target of ^18^F-FLT and ^18^F-FDG with GTV_P_. The p-value is measured in compare with the pathological GTV.

## Abbreviations

NPC: Nasopharyngeal carcinoma
SEER: Surveillance, epidemiology, and end results
MRI: Magnetic resonance imaging
CT: Computed tomography
PET: Positron emission tomography
GTV: Gross tumor volume
^18^F-FLT: ^18^F-Fluorothymidine
^18^F-FDG: ^18^F-Fluorodeoxyglucose
NIH: National Institutes of Health
SUV: Standardized uptake value
SD: Std. deviation
