# Supplementary material for: Comparison of different images in gross target volume delineating on VX2 nasopharyngeal transplantation tumor models
Source: J Cancer. 2020 Jan 1;11(5):1104–14. doi: 10.7150/jca.36076 (PMC6959086; doi:10.7150/jca.36076)

SUPPLEMENTAL MATERIAL

Title:

Comparison of different images in gross target volume delineating on VX2 nasopharyngeal transplantation tumor models

Inventory of Supplemental Information

- Supplemental Fig. 1 (Page 1)

- Supplemental Fig. 2 (Page 2)

Figure S1. The different SUV threshold values on ^{18}F-FLT PET/CT images. "*" represents no statistically significant difference of GTV_{FLT} in compared with GTVp ($P > 0.05$).

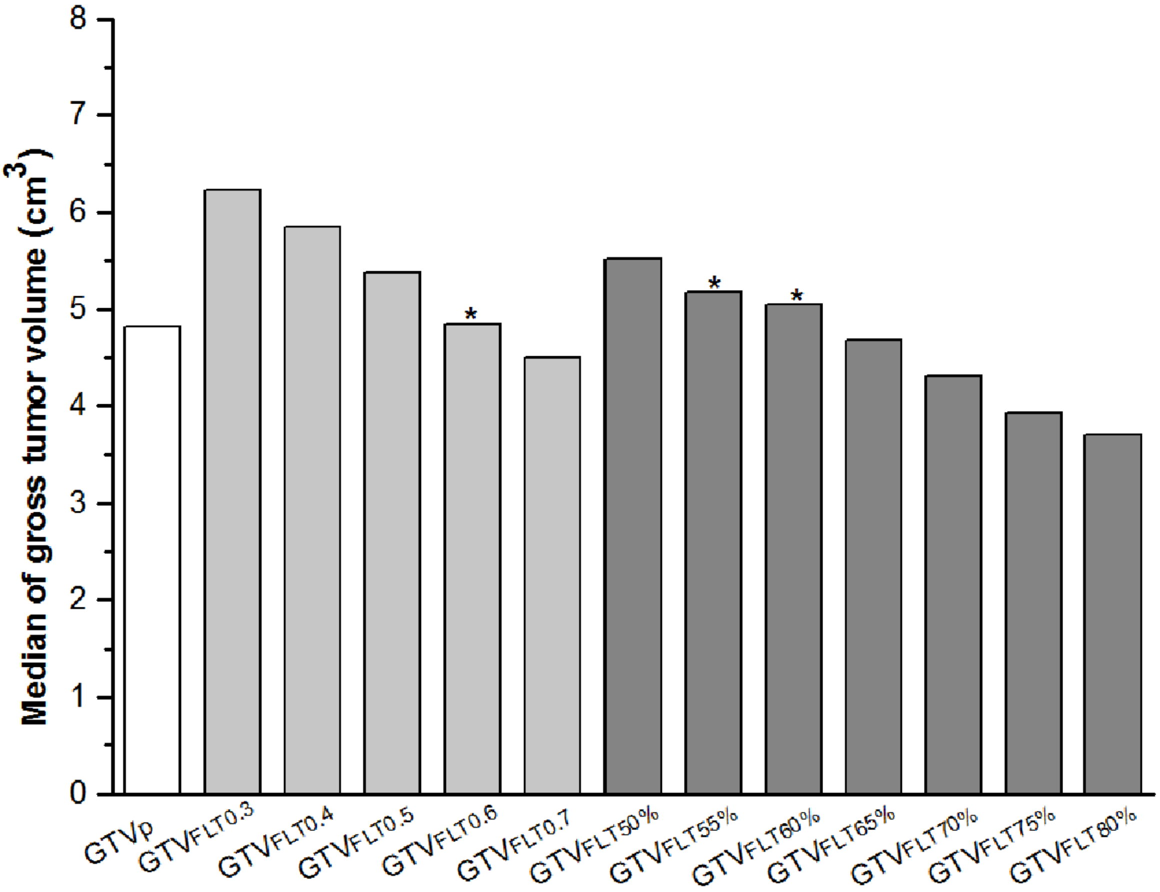

Figure S2. The different SUV threshold values on ^{18}F-FDG PET/CT images. “*” represents no statistically significant difference of GTV_{FDG} in compared with GTVp ($P > 0.05$).

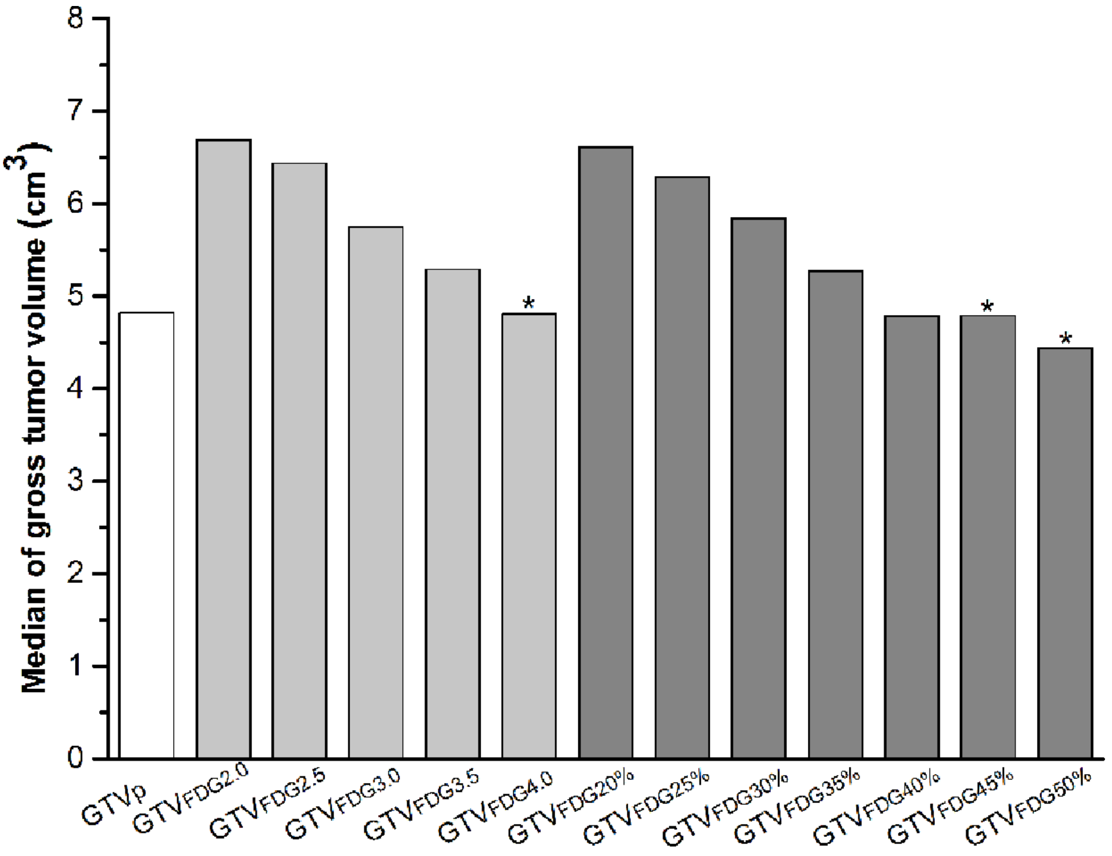

Supplement: Supplementary file 1 — Supplementary figures. [file jcav11p1104s1.pdf]
